# Non-targeted proteomic analysis of Asian elephant (*Elephas maximus*) seminal plasma using an in-solution digestion technique and liquid chromatography tandem-mass spectrometry

**DOI:** 10.3389/fvets.2023.1174078

**Published:** 2023-09-20

**Authors:** Podjana Wattananit, Yodying Yingchutrakul, Kornchai Kornkaewrat, Sittidet Mahasawangkul, Sittiruk Roytrakul, Anuchai Pinyopummin

**Affiliations:** ^1^Faculty of Veterinary Science, Mahidol University, Nakhon Pathom, Thailand; ^2^National Center for Genetic Engineering and Biotechnology (BIOTEC), National Science and Technology Development Agency, Pathum Thani, Thailand; ^3^Faculty of Veterinary Medicine, Kasetsart University, Nakhon Pathom, Thailand; ^4^The National Elephant Institute, The Forest Industry Organization, Lampang, Thailand

**Keywords:** Asian elephant, seminal plasma, protein, proteomic analysis, male reproduction

## Abstract

Seminal plasma proteins have recently been reported to play a significant role as valuable materials for understanding male reproductive biology, identifying causes of fertility problems, and developing reproductive biomarkers. Proteomic analysis of seminal plasma holds promise in advancing the understanding of male Asian elephant reproductive biology. This study aims to explore seminal plasma proteins of Asian elephants and their probable functions to provide fundamental information about male reproduction in this species. The protein solution from pooled seminal plasma from 10 bulls (a total of 33 ejaculates) was digested into peptides and identified using LC-MS/MS. Out of 986 proteins, 597 were mapped and matched with 58 species in UniProt databases, including *Elephas maximus*. These mapped proteins were mostly involved in binding function, catalytic activity, cellular process, and metabolic process. Only 29 mapped proteins were recognized to be related in reproductive process, mainly associated in spermatogenesis and sperm capacitation. Additionally, several seminal plasma proteins related to fertility or semen quality in other mammals were also found in Asian elephant semen, such as keratin type I, aldose reductase, thrombospondon-1, fibronectin 1, platelet-activating factor acetyl hydrolase, mannosidase, and semenogelin-2. This discovery clearly reveals the beneficial protein profile in seminal plasma of the Asian elephant and serves as a crucial step in investigating infertility and poor semen quality in this valuable species.

## Introduction

The International Union of Conservation of Nature (IUCN) currently lists the Asian elephant (*Elephas maximus*) as endangered in the Red List of Threatened Species. This designation is due to the declining numbers of the Asian elephant population, even in captivity. A major contributing factor to the decline of the captive population of Asian elephant is the low birth rate (1, 2). In terms of male reproductive factors, the quality and quantity of semen have a significant impact on fertility and the success of artificial insemination (AI). The inconsistency of semen quality observed following collection using the per rectum massage technique necessitates comprehensive investigation to determine the underlying causes and develop potential strategies for improvement (3).

Seminal plasma (SP), consisting of a plentiful number of proteins in terms of quality and quantity, is a combined fluid secreted from the testis, epididymis, and accessory sex glands in male mammals (4). SP, which accounts for approximately 90% of semen volume, has been reported as an important medium affecting sperm quality and fertilizing ability (5). SP proteins have been reported to have an essential influence on preserving sperm survival, sperm capacitation, and sperm-oocyte binding during fertilization in the female reproductive tract (6). Over the last decade, SP proteins have been extensively investigated for their influence on male fertility/subfertility and sperm function in various species, aiming to develop biomarkers for early detection of fertility/subfertility (6–11). In human, certain proteins found in seminal plasma can potentially be used as biomarkers to identify patients with oligozoospermia, abnormal morphology, or infertility (9). Furthermore, gaining knowledge about SP proteins can lead to a better understanding of reproductive physiology and assisting in the development of new storage protocols (6).

There have been very few studies on SP proteins in Asian elephants. The relationship between the amount of protein in Asian elephant SP and sperm motility has been explored in published studies. However, the results from these publications present contrasting findings. One study demonstrated a positive correlation between protein levels and sperm motility (12), while another study indicated the opposite (13). Interestingly, the protein lactotransferrin appears to serve as an indicator of high sperm motility in this species. It is noteworthy that lactotransferrin is only present in 85% of semen samples exhibiting high motility (≥65% sperm motility), while it is absent in 90% of semen samples with poor motility (≤10% sperm motility) (13). The composition of proteins in SP seems to play a more significant role in sperm biology than the overall volume of proteins. These findings strongly support further investigation into the complete protein profile of seminal plasma in Asian elephants (14), which would provide valuable insights into the types and functions of proteins involved.

Proteomic analysis is a useful tool for the identification of protein characteristics and functions, including protein interactions. This assessment has been significant in exploring the nature of male reproduction, such as proteomics of seminal plasma and spermatozoa (8, 9). Recently, the mass spectrometric (MS) technique has been widely applied for protein identification, even in complex samples such as seminal plasma (15). Protein mass spectrometry can be broadly categorized into two approaches: untargeted and targeted proteomics. Untargeted or non-targeted proteomics aims to discover the near-complete proteome coverage, which involves the identification and/or quantification of a maximum number of proteins possible (16). Typically, protein identification, the allocation of covalent modifications, the detection of sequence errors, and even *de novo* sequencing are commonly performed at the peptide level. The choice of digestion method and its conditions must be meticulously made, taking into account both the protein sequence and the desired information to be extracted. In order to have greater influence on the results of the procedure, in-solution digestion is favored over in-gel digestion (17). In-solution digestion offers greater automation potential and reduces the need for extensive sample manipulation (18).

The objective of this proteomic analysis conducted on seminal plasma from Asian elephants was to investigate the complete protein composition and their respective functions. The in-solution digestion technique and liquid chromatography tandem-mass spectrometry (LC-MS/MS) were employed for this purpose. The identification of this protein profile is anticipated to assist future research on male reproductive issues, including challenges related to semen quality and infertility in this particular species.

## Materials and methods

### Semen collection and analysis

The animal care and use protocol was approved by the Institutional Animal Care and Use Committee, Kasetsart University (ACKU61-VET-014). Semen was collected every month using a transrectal massage technique (19) from 10 sexually mature elephant bulls, ranging in age from 15 to 60 years old. These elephants were housed at the National Elephant Institute (NEI), Forest Industry Organization, located in Lampang, Thailand (latitude and longitude: 18° 5.2 m 134.6 cm N/99° 30′ 63.5 cm E) during January 2014–December 2016. Collecting semen and conducting basic semen evaluation were regular tasks involved in the breeding management of elephants at this organization by the NEI staff. The total of 33 ejaculates without urine contamination from 10 bulls (three ejaculates from seven bulls and four ejaculates from three bulls) was included in the study. The semen quality of each ejaculate was evaluated for volume, pH, sperm concentration, motility, viability, and sperm morphology. Sperm concentration was measured using a hemocytometer (Paul Marienfeld GmbH & Co.KG, Lauda-Königshofen, Germany), while the percentage of sperm motility was estimated under a phase-contrast microscope (Olympus (Thailand) Co., Ltd., Bangkok, Thailand) at a magnification of ×200. A standardized 10 μL drop of semen was pipetted onto a pre-warmed (38°C) glass slide and covered with a coverslip for examination. Sperm viability evaluation was conducted using eosin-negrosin technique (20). For this, 200 spermatozoa per sample were examined, with unstained sperm classified as live and stained sperm classified as dead. Sperm morphology was assessed using William’s staining according to the procedure developed by Sarder (21). Smears of fresh semen were air-dried, fixed in absolute alcohol for 3 min, and stained with William’s stain for 5 min. After staining, the slides were washed in running tap water, dried, and examined using bright-field light microscopy at a magnification of ×1,000. Again, 200 spermatozoa per sample were examined for head, mid-piece, and tail abnormalities, or classified as having normal morphology.

### Seminal plasma preparation for protein analysis

One mL of fresh semen was mixed with 10 μL of a protease inhibitor cocktail (Sigma-Aldrich) (11) and immediately centrifuged at 700 g 4°C for 15 min. After centrifugation, the seminal plasma from the supernatant was pipetted into a new tube. Samples were then centrifuged at 10,000 g 4°C for 1 h to ensure the removal of sperm cells and debris from the seminal plasma. The seminal plasma was kept at −20°C for protein analysis. To create a pooled sample, 50 μL of thoroughly mixed seminal plasma from each ejaculate of 33 ejaculates of Asian elephant bulls was combined in a sterile 5-mL tube. Similarly, for each individual bull sample, 50 μL of well-mixed seminal plasma from each ejaculate was combined in a separate sterile 1.5-mL centrifuge tube, resulting in a total of 10 individual samples for 10 bulls. The pooled and individual seminal plasma samples were measured for total protein and prepared for further protein identification of Asian elephant seminal plasma.

Total protein in seminal plasma was determined using Lowry’s method (22) with bovine serum albumin (Sigma-Aldrich) as standards. Five μg of seminal plasma proteins were dried in a low-binding microcentrifuge tube using a speed vacuum centrifuge for 30 min. The dried samples were then resuspended with 10 μL of 10 mM Ammonium bicarbonate (Ambic). Seminal plasma proteins in Ambic were digested into peptides following an in-solution digestion protocol modified from Leon et al. (23). Briefly, the protein solutions were reduced with 10 mM Dithiothreitol (DTT) in 10 mM Ambic at 56°C for 1 h. After allowing the samples to cool at room temperature, the protein samples were alkylated with 30 mM Iodoacetamide (IAA) in 10 mM Ambic at room temperature in the dark for 1 h. The next step involved digesting the protein solutions using 0.25 μg trypsin (1 μg trypsin per 20 μg protein) at 37°C overnight. Samples were then spun down at 10,000 g for 30 s and dried using a speed vacuum centrifugation. The dry samples were frozen at −20°C until analysis with LC-MS/MS.

### Protein quantitation and identification

The digested peptides were analyzed in an Ultimate3000 Nano/Capillary LC System (Thermo Scientific, Massachusetts, United States) coupled to a Q-Tof impact II™ (Bruker Daltonics, Massachusetts, United States) equipped with a Nano-captive spray ion source. One hundred ng of the digested peptide was subjected to the trapping column (Thermo Scientific, PepMap100, C18, 300 μm i.d. × 5 mm), using full loop injection. The sample was resolved on an analytical column (PepSwift Monolithic Nano Column, 100 μm × 5 cm i.d.) at column temperature of 60°C. The mobile phase A and B were 0.1% (v/v) formic acid in water and 80% (v/v) acetonitrile in 0.1% (v/v) formic acid, respectively. Peptide separation was accomplished under gradient conditions of 1%–60% B over 10 min at a flow rate of 1 μL/min. Electrospray ionization was carried out at 1.6 kV using the CaptiveSpray. Mass spectra (MS) and MS/MS spectra were fully acquired in the positive-ion mode over the range m/z 150–2,200 (Compass 1.9 for otofSeries software, Bruker Daltonics). Mass accuracy in positive detection mode after tune/internal calibration with Sodium trifluoroacetate (NaTFA) was within 1.6 ppm. LC-MS/MS spectra were acquired using a data-dependent auto-MS/MS method, with a dynamic method, and a fixed cycle time of 3 s. DeCyder MS Differential Analysis software (DeCyderMS, GE Healthcare, Illinois, United States) (24, 25) was used for protein quantitation and raw data from DeCyder were searched against the mammal protein database from NCBI using the MASCOT (Matrix Science, London, United Kingdom) (26) search engine for protein identification. Searches were performed with a maximum of three missed cleavages, carbamidomethylation of Cys as a fixed modification, and oxidation of Met as variable modifications. The level of proteins in each sample were expressed as log2 value. The MS/MS raw data and analysis files have been deposited in the ProteomeXchange Consortium^1^ via the jPOST partner repository^2^ with the data set identifier JPST002020 and PXD039856.

### Data and gene ontology analysis

Statistical analysis of semen quality parameters was carried out in the R (version 3.5.1, R foundation). The results are presented as the means and standard deviation (mean ± SD). Protein data after the MASCOT search was identified using UniProt protein database (27). Gene ontology analysis using UniProt via https://www.uniprot.org/id-mapping (for only reproductive process) and the Protein Analysis Through Evolutionary Relationship (PANTHER) online tool^3^ (28) was performed for functional classification of UniProt mapped proteins. The protein–protein interactions (PPI) were explored using STRING database via https://string-db.org/ (29). For further investigation, the SP proteins were searched to determine their recognition in previous publications on human and other male animal research. This was done by using their names as search terms through platforms such as Google Scholar and PubMed. The analysis was conducted using all these database sources accessed between November 2022–June 2023.

## Results

The average (mean ± SD) age of 10 elephant bulls in this experiment was 32.55 ± 10.09. The semen quality of each ejaculate is presented in Table 1. The average percentages (mean ± SD) of sperm motility, viability, and normal morphology were 34.39 ± 26.54, 47.09 ± 30.70, and 72.76 ± 25.88, respectively.

**Table 1:** Semen parameters of Asian elephant bulls: assessing sperm motility, viability, morphology, concentration, volume, and pH in 33 ejaculates from 10 individuals collected during January 2014–Dec 2016 at NEI, Lampang, Thailand.

Elephant Id	No. of ejaculation	% Motility	% Viability	% Normal morphology	Sperm concentration (cells × 10^6^/mL)	Volume (mL)	pH
E2	1	30	30	87	950	1	8.5
	2	40	82	81.5	1,580	23	7.5
	3	60	77	95	1,640	32	7
E3	1	60	92	92	2,865	20	7
	2	40	78	79	1,573	13	7
	3	50	83	82	899	16	7
E4	1	80	92	78.5	1,695	35	8
	2	40	61	70.5	1,485	120	7.5
	3	5	26	59	995	8	8
	4	50	62	74	1785	30	7.5
E5	1	30	30	47	1,040	1	8
	2	50	52	97.5	2,585	12	5.5
	3	5	12	19	900	26	8
	4	0	10	16.5	775	8	5
E6	1	60	78	91	1790	0.9	7.5
	2	70	72	91	815	16	7
	3	5	12	94	786	5	6.5
	4	50	65	97	1,456	50	6
E8	1	70	81	97	1,270	33	8.5
	2	80	77	93.5	3,000	20	7
	3	60	73	96.5	1,540	17	8
E9	1	10	12	85.5	575	17	8
	2	5	10	84	1,370	30	7.5
	3	0	10	82.5	361	4.5	8
E10	1	30	45	95	2,455	24	6
	2	0	0	15.5	690	11	5.5
	3	50	70	93	1,387	24	7
E11	1	10	10	50.5	775	9	7
	2	0	12	30	287	10.5	9
	3	25	36	87	1,200	1.5	8
E13	1	60	70	56.5	1,460	38	6
	2	5	22	43	540	10	7
	3	5	12	40	396	35	8
Mean ± SD		34.39 ± 26.54	47.09 ± 30.70	72.76 ± 25.88	1300.61 ± 690.21	21.26 ± 21.61	7.24 ± 0.95

**Figure 1:**
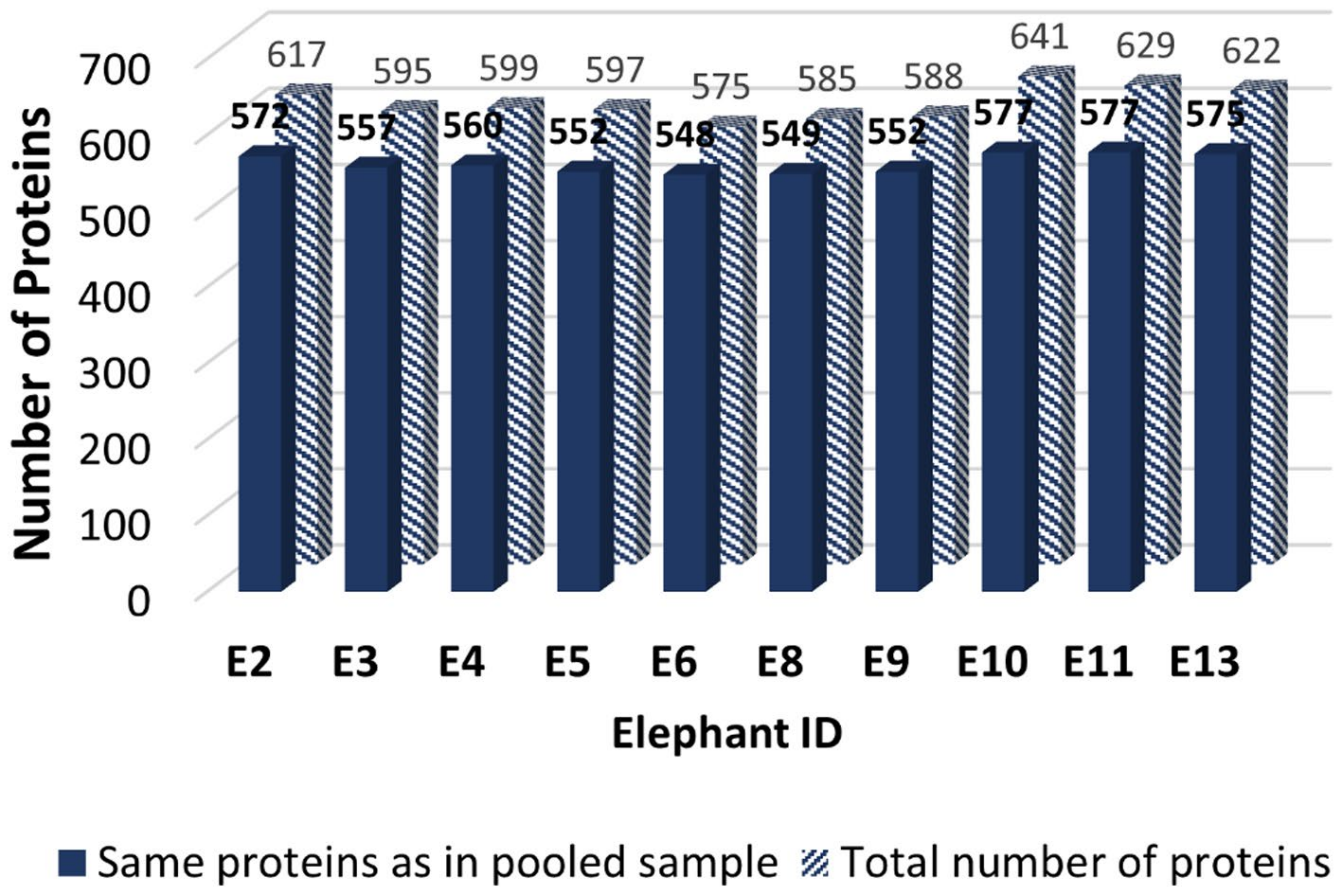
The bar graph illustrating the quantity of mapped Asian elephant SP proteins obtained from Uniprot database of seminal plasma samples from a cohort of 10 male elephants: E2, E3, E4, E5, E6, E8, E9, E10, E11, and E13. The solid bar represents the SP proteins that are common to both individual samples and the pooled sample from all bulls, whereas the striped bar indicates the total number of SP proteins found in the individual samples.

The LC-MS/MS revealed 1,183 proteins from seminal plasma, which included both the pooled sample and 10 individual samples (Supplementary Table 1). From the pooled seminal plasma sample, 986 proteins were identified using LC-MS/MS. However, only 597 SP proteins from the pooled sample were matched in the UniProt databases (Supplementary Table 2). Similarly, the total number of mapped proteins in the 10 individual samples (E2, E3, E4, E5, E6, E8, E9, E10, E11, and E13) was approximately 605. Figure 1 illustrates the quantity of the same SP proteins found in individual samples compared to the pooled sample. The proportion of equivalent proteins between the individual samples and in the pooled sample for E2, E3, E4, E5, E6, E8, E9, E10, E11, and E13 was calculated as a percentage: 92.71%, 93.61%, 93.49%, 92.46%, 95.30, 93.85%, 93.88%, 90.02%, 91.73%, and 92.44%, respectively. Based on these results, the mapped proteins’ profile from the pooled sample was considered representative of all individual samples for further analysis. Out of a total of 58 species that matched with 597 SP proteins in the pooled sample, including *Elephas maximus*, most of them were recognized in *Homo sapiens* (160 proteins), *Mus musculus* (140 proteins), *Rattus norvegicus* (48 proteins), *Bos Taurus* (34 proteins), and *Ailuropoda melanoleuca* (25 proteins). Among those matched proteins in Uniprot database, only two SP proteins were identified as belonging to the Asian elephant species in UniProt: serum albumin (ESA or ALB) and trace amine-associated receptor 4 (TAAR4).

**Figure 2:**
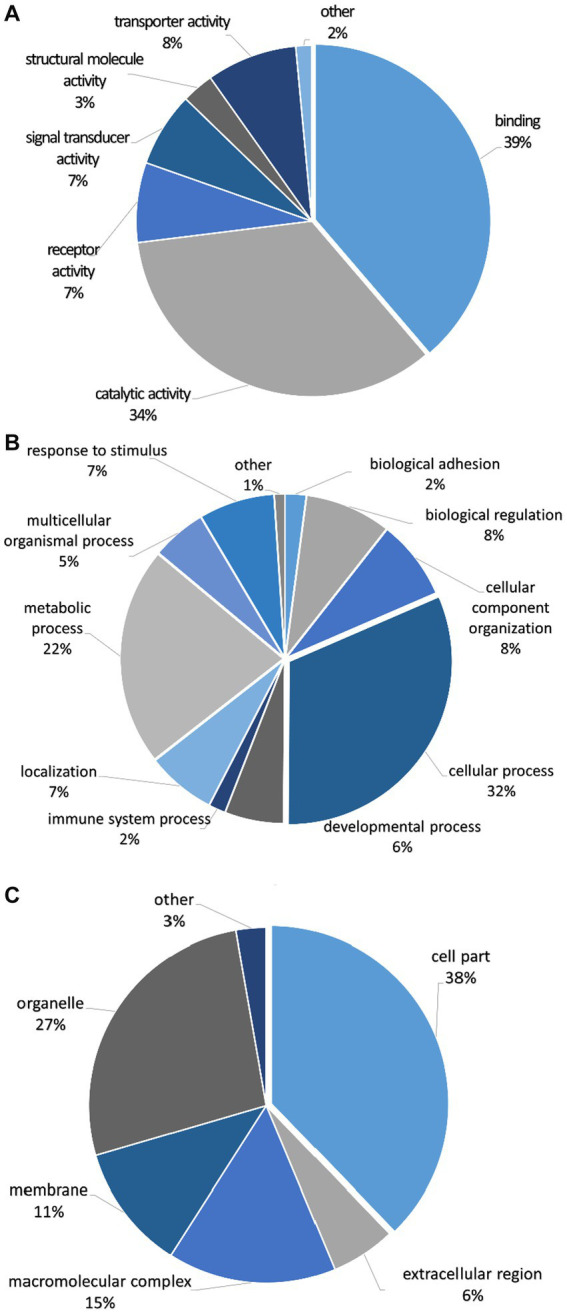
Classification of seminal plasma proteins from Asian elephant semen in terms of **(A)** molecular function, **(B)** biological process, and **(C)** cellular component using PANTHER as a gene ontology analysis. The quantities of proteins in each class are presented as percentages in pie charts.

**Figure 3:**
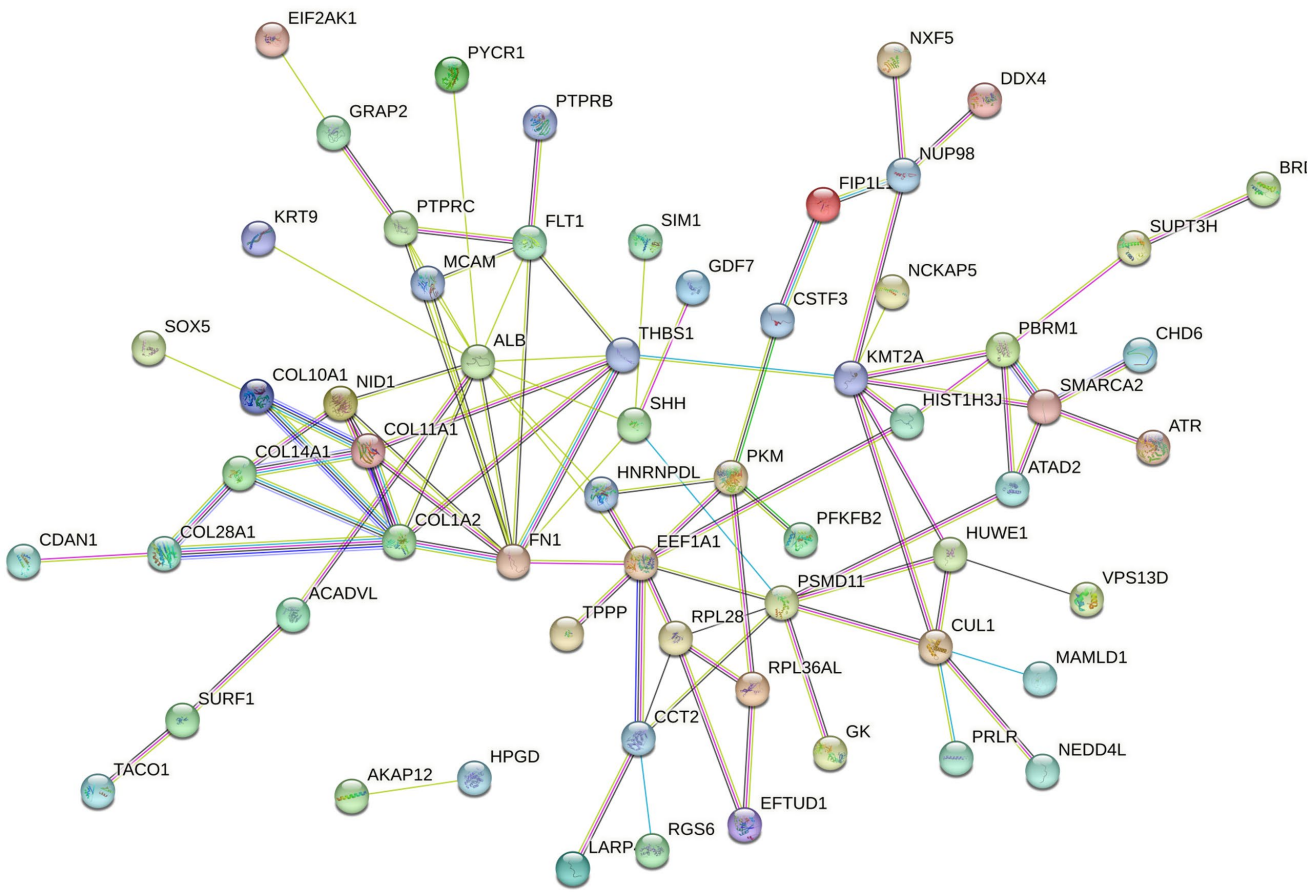
The protein–protein interaction (PPI) network of mapped Asian elephant SP proteins, constructed using data from the STRING database, was recognized in *Homo sapiens* (human). Sixty-one SP proteins displayed interactions among them at different levels. The different types of associations are presented in different colors of the linkage lines between proteins: light blue for curated database interactions, pink for experimental interactions, green for gene neighborhoods, red for gene fusions, blue for gene co-occurrence, light green for text-mining, black for co-expression, and purple for protein homology.

**Figure 4:**
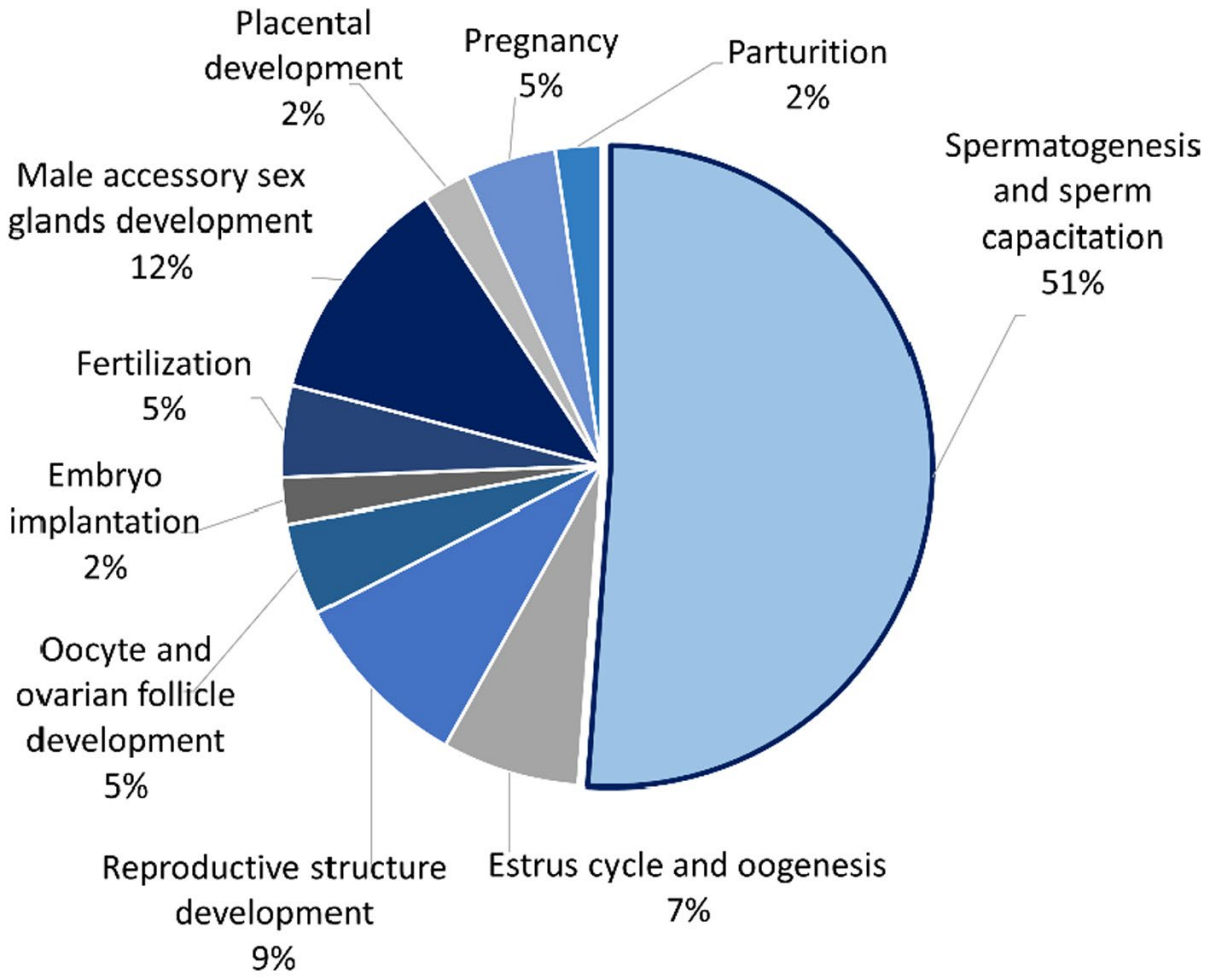
Pie chart showing percentage of seminal plasma proteins from Asian elephant semen divided into several functions that are involved in the reproductive process using UniProt as a gene ontology analysis.

The mapped proteins from UniProt were then classified separately based on molecular function, biological process, and cellular component (Figure 2), as well as biological pathway (Supplementary Table 3), using PANTHER. A total of 204 hits of mapped proteins were categorized based on molecular function (Figure 2A), with the majority of them being involved in binding function (39%) and catalytic activity (34%). In terms of biological process classification (Figure 2B), there were 481 hits, primarily associated with cellular process (32%) and metabolic process (22%) function. Additionally, within the cellular component category (Figure 2C), the majority of proteins from 254 hits were grouped as cell parts (38%) and organelles (27%). Sixty-one SP proteins of the Asian elephant, which were recognized as human proteins in the STRING database, were found to have interactions among themselves. These proteins are associated with various cellular processes based on their biological functions, except for the Mastermind-like domain-containing protein 1 (MAMLD1). These proteins include ALB, fibronectin 1 (FN1), Thrombospondin-1 (THBS1), sonic hedgehog protein (SHH), Elongation factor 1-alpha 1 (EEF1A1), Tubulin polymerization-promoting protein (TPPP), T-complex protein 1 subunit beta (CCT2), probable ATP-dependent RNA helicase DDX4 (DDX4), Nuclear pore complex protein Nup98-Nup96 (NUP98), probable global transcription activator SNF2L2 (SMARCA2), serine/threonine-protein kinase (ATR), and others. There were 52 pairs of these proteins showing PPI as gene co-expression such as ALB and FN1, EEF1A1 and TPPP, EEF1A1 and CCT2, and others (Figure 3). Interestingly, gene ontology analysis conducted in UniProt revealed that out of 29 SP proteins, 44 hits were related to reproductive process, with a significant involvement in spermatogenesis and sperm capacitation (51%; Figure 4). The 29 SP proteins related to reproductive processes were presented in Table 2, along with an example of their specific reproductive functions. For instance, BTB/POZ domain-containing protein 18 (BTBD18), DDX4, SMARCA2, Spermatogenesis-associated protein 18 (SPATA18), Spermatogenesis-associated protein 32 (SPATA32), and Zinc finger protein 105 (ZFP105) have been reported to be associated with spermatogenesis.

**Table 2:** The list of reproductively associated SP proteins in Asian elephants (based on 33 ejaculates from 10 bulls) using Uniprot gene ontology.

UniProt code	Protein name	Recognized species	Example of function in reproductive process
P15428	15-hydroxyprostaglandin dehydrogenase (HPGD)	*Homo sapiens* (Human)	Parturition
P51647	Aldehyde dehydrogenase family 1 member A1 (AlDH1A1)	*Rattus norvegicus* (Rat)	Estrous cycle
P22086	Alpha-2C adrenergic receptor (ADRA2C)	*Rattus norvegicus* (Rat)	Female pregnancy
F6YBC0	Beta-1,3-N-acetylglucosaminyltransferase (LFNG)	*Monodelphis domestica* (Gray short-tailed opossum)	Meiotic cell cycle process
A0A0A6YY25	BTB/POZ domain-containing protein 18 (BTBD18)	*Mus musculus* (Mouse)	Spermatogenesis
G1MHQ3	Cation channel sperm associated 3 (CATSPER3)	*Ailuropoda melanoleuca* (Giant panda)	Sperm capacitation
Q8BRT1	CLIP-associating protein 2 (CLASP2)	*Mus musculus* (Mouse)	Meiotic cell cycle
Q96CW5	Gamma-tubulin complex component 3 (TUBGCP3)	*Homo sapiens* (Human)	Single fertilization
H2PH52	Glycine receptor alpha 1 (GLRA1)	*Pongo abelii* (Sumatran orangutan)	Acrosome reaction
Q7Z4P5	Growth/differentiation factor 7 (GDF7)	*Homo sapiens* (Human)	Reproductive structure development
P23441	Homeobox protein Nkx-2.1 (NKX2-1)	*Rattus norvegicus* (Rat)	Development of primary female sexual characteristics
P14616	Insulin receptor-related protein (INSRR)	*Homo sapiens* (Human)	Male sex determination
P11679	Keratin, type II cytoskeletal 8 (KRT8)	*Mus musculus* (Mouse)	Cell differentiation in embryonic placenta development
O88978	Leucine-rich repeat-containing protein 6 (LRRC6)	*Mus musculus* (Mouse)	Male gonad development
O88572	Low-density lipoprotein receptor-related protein 6 (LRP6)	*Mus musculus* (Mouse)	External genitalia morphogenesis
Q13495	Mastermind-like domain-containing protein 1 (MAMLD1)	*Homo sapiens* (Human)	Male gonad development
Q8IZA3	Oocyte-specific histone H1 (H1FOO)	*Homo sapiens* (Human)	Meiotic cell cycle
P63005	Platelet-activating factor acetylhydrolase IB subunit alpha (PAFAH1B1)	*Mus musculus* (Mouse)	Acrosome assembly
Q9NQI0	Probable ATP-dependent RNA helicase DDX4 (DDX4)	*Homo sapiens* (Human)	Spermatogenesis
P51531	Probable global transcription activator SNF2L2 (SMARCA2)	*Homo sapiens* (Human)	Spermatid development
P16471	Prolactin receptor (PRLR)	*Homo sapiens* (Human)	Embryo implantation
Q5U7N4	Semenogelin-2 (SEMG2)	*Pan troglodytes* (Chimpanzee)	Sperm capacitation
Q15465	Sonic hedgehog protein (SHH)	*Homo sapiens* (Human)	Prostate gland development
Q0P557	Spermatogenesis-associated protein 18 (SPATA18)	*Mus musculus* (Mouse)	Spermatogenesis
Q8C5V0	Spermatogenesis-associated protein 32 (SPATA32)	*Mus musculus* (Mouse)	Spermatogenesis
P78371	T-complex protein 1 subunit beta (CCT2)	*Homo sapiens* (Human)	Binding of sperm to zona pellucida
Q8C2E7	WASH complex subunit 5 (KIAA0196)	*Mus musculus* (Mouse)	Oocyte maturation
Q5M881	Zinc finger protein 105 (ZFP105)	*Rattus norvegicus* (Rat)	Spermatogenesis

It was found that 34 of the proteins detected in Asian elephant seminal plasma shared similarities with SP proteins in various species, including boar, bull, ram, and human (Table 3). It is worth noting that the proteins KRT9, Platelet-activating factor acetylhydrolase IB subunit alpha (PAFAH1B1), Semenogelin-2 (SEMG2), SPATA18, SPATA32, and T-complex protein 1 subunit beta (CCT2), which were identified as SP proteins related to reproductive processes in Table 2, are also included in Table 3.

**Table 3:** Comparative analysis of SP proteins in Asian elephants: identifying homologous proteins from previous studies assessing male reproductive performance in other species such as *Sus scrofa domesticus* (boar), *Bos taurus* (cattle bull), *Bos indicus* (Guzerat bull), *Ovis aries* (ram), and *Homo sapiens* (human).

SP proteins in Asian elephants	SP proteins in other species		
	Protein name	Species	References
Aldose reductase-related protein 1 (AVDP)	Aldose reductase (AKR1B1)	*Sus scrofa domesticus*	(4)
Alpha-1,2-mannosidase (MAN1C1)	Predicted: alpha mannosidase 2C1 (MAN2C1)	*Bos taurus*	(7) (11)
	Beta mannosidase (MANB1)	*Ovis aries*	
Bactericidal/permeability-increasing protein-like 3 (BPIFB6)	Bactericidal/permeability-increasing protein (BPI)	*Ovis aries*	(11)
Beta-defensin 107 (DEFB107)	Predicted: beta-defensin 1 (DEFB1)	*Bos taurus*	(7)
Chaperone Hsp 60 kDa (HSP60)	Chaperone Hsp 70 kDa (HSP70)	*Bos taurus*, *Ovis aries*	(7, 11)
	Chaperone Hsp 90 kDa (HSP90)	*Bos taurus*	(7)
Cilia- and flagella-associated protein 74 (CFAP74)	Cilia- and flagella-associated protein 70 (CFAP70)	*Ovis aries*	(30)
Coiled-coil domain-containing protein 66 (CCDC66)	Coiled-coil domain-containing protein (CCDC)	*Ovis aries*	(30)
Coiled-coil domain-containing protein 89 (CCDC89)			
Coiled-coil domain-containing protein 105 (CCDC105)			
Dynein heavy chain 3, axonemal (DNAH3)	Dynein heavy chain 2, axonemal (DNAH2)	*Ovis aries*	(30)
Fibronectin 1 (FN1)	Fibronectin 1 (FN1)	*Sus scrofa domesticus*	(31)
Hypothetical protein LOC507206 (TIMMDC1)	Hypothetical protein LOC512373	*Bos taurus*	(7)
Immunoglobulin gamma 1 chain C region (IGHG1)	Immunoglobulin gamma 1 chain C region (IGHG1)	*Bos taurus*	(7)
	Immunoglobulin gamma 2 chain C region (IGHG2)		
	Immunoglobulin lambda light chain (IGLL)		
Keratin, type I cytoskeletal 9 (KRT9)	Keratin, type I cytoskeletal 17 (KRT17)	*Sus scrofa domesticus*	(4)
Keratin, type I cytoskeletal 15 (KRT15)			
Lamin B2 (LMNB2)	Lamin A/C (LMNA)	*Ovis aries*	(30)
Mesothelin (MSLN)	Predicted: mesothelin isoform 1 (MSLN1)	*Bos taurus*	(7)
Nuclear pore complex protein Nup98-Nup96 (NUP98)	Nuclear pore complex protein Nup155 (NUP155)	*Ovis aries*	(30)
Phosphoglycerate kinase (PGK2)	Predicted: phosphoglycerate kinase, testis-specific	*Bos taurus*	(7)
	Phosphoglycerate kinase 2 (PGK2)	*Ovis aries*	(9)
Platelet-activating factor acetylhydrolase IB subunit alpha (PAFAH1B1)	Platelet-activating factor acetylhydrolase (PAFA)	*Bos tauru*, *Bos indicus*	(7, 32)
Protein FAM171B (FAM171B)	Protein FAM154A (FAM154A)	*Ovis aries*	(30)
Protein FAM186A (FAM186A)			
Semenogelin 2 (SEMG2)	Semenogelin 2 (SMEG2)	*Homo sapiens*	(33)
Serum albumin (ALB or ESA)	Serum albumin (ALB)	*Bos taurus*	(7)
Spermatogenesis-associated protein 18 (SPATA18)	Spermatogenesis-associated protein 32 (SPATA32)	*Ovis aries*	(30)
Spermatogenesis-associated protein 32 (SPATA32)			
T-complex protein-1, beta subunit (CCT2)	Predicted: T-complex protein-1 (TCP1), alpha subunit	*Bos taurus*	(7)
	Predicted: T-complex protein-1 (TCP1), epsilon subunit, isoform 1, 2 or 3		
	Predicted: T-complex protein-1 (TCP1), eta subunit		
	Predicted: T-complex protein-1 (TCP1), gamma subunit		
Testis-expressed protein 33 (TEX33)	Predicted: testis expressed sequence 101 (TEX101)	*Bos taurus*	(7)
Testis-expressed protein 35 (TEX35)			
Thrombospondin-1 (THBS1)	Thrombospondin-1 (THBS1)	*Sus scrofa domesticus*	(4)
Thrombospondin type-1 domain-containing protein 1 (THSD1)			
Tubulin epsilon and delta complex 2 (TEDC2)	Tubulin, beta-2 (TUBB2)	*Bos taurus*	(7)
Tubulin polymerization-promoting protein (TPPP)	Tubulin polymerization-promoting protein 2 (TPPP2)	*Ovis aries*	(9)
Very long-chain specific acyl-CoA dehydrogenase, mitochondrial (ACADVL)	Medium-chain specific acyl-CoA dehydrogenase, mitochondrial (ACADM)	*Ovis aries*	(30)

## Discussion

The inconsistency of semen quality, especially sperm motility, among ejaculates within each bull was observed in Asian elephant semen in this experiment. This finding is similar to previous reports (3, 13, 14). In this case, conducting proteomic analysis on the pooled semen sample that includes ejaculates with both poor and high quality from all bulls may provide a more reliable assessment of the whole proteomes in the seminal plasma of this species.

Based on the gene ontology analysis, the functional analysis of seminal plasma (SP) proteins in Asian elephants showed similar results to those described in bovine (34) and buffalo SP proteome (35). The highest proportion of SP proteins in terms of molecular function was found to be associated with binding, accounting for 39% in Asian elephants, 44% in bovines, and 60% in buffaloes. Similarly, the highest proportion of SP proteins related to cellular processes was observed in the biological process category, with percentages of 32%, 24%, and 60% in Asian elephants, bovines, and buffaloes, respectively. In many instances, the protein-binding function observed in seminal proteins may serve as a supporting part to their primary function such as enzymatic and transport activities. They were reported to be bound to the sperm surface during ejaculation, leading to the formation of protein-coating layers (36).

In humans, the primary category of seminal proteins consists of various enzymes involved in catalytic activities, making up approximately 33%–65% of the total protein composition. Additionally, around 5%–14% of the SP proteins are categorized as regulators of these enzymes (36). Similarly, boar SP proteins were reported to have 41% proteins categorized as catalytic activities (4). However, human SP proteins in some publications revealed the most commonly reported annotation found in seminal proteins is binding activity (37, 38). Some mapped SP proteins of the Asian elephant involved in cellular processes also exhibited protein–protein interactions within their functional group. ALB and FN1 have been suggested as potential biomarkers for oxidative stress in terms of male infertility factors (39). This report confirms the potential interaction between these protein types (39). However, further investigation is required to understand the interactions within the group of SP proteins in this species. According to the search results from the STRING database, they were mentioned together in several publications as text-mining interactions. Nonetheless, their interactions in relation to reproductive biology remain uncertain.

ESA and TAAR4 are only two matched SP proteins, which were recognized as Asian elephant proteins in the database. ESA has been reported to be a shuttle of pheromones in Asian elephants. There is substantial evidence confirming that the ESA protein binds with pheromones in the elephant bloodstream and is subsequently transported into urine. A considerable portion of the pheromone present in urine is found to be bound to ESA as a complex (40). While TAARs were recognized as a distinct group of G-protein-coupled receptors with a specific responsiveness to trace amines rather than classical biogenic amines. The TAAR family in mammals comprises a total of nine subtypes. In mice, the olfactory epithelium expresses all TAAR subtypes, except for TAAR1, which act as chemosensory receptors for volatile amines (41). In recent times, there has been a proposition that murine TAAR3, TAAR4, and TAAR5 are involved in chemosensory functions related to the recognition of volatile amines. Interestingly, humans can still detect the scent of volatile amines, even though they have disruptions in the open reading frames (ORFs) of TAAR3 and TAAR4 (42). No evidence has been found yet regarding the functional analysis of TAAR4 in Asian elephants.

Based on the gene ontology analysis conducted with the UniProt database, several Asian elephant SP proteins were prominently linked to spermatogenesis such as BTBD18, DDX4, SMARCA2, SPATA18, SPATA32, ZFP105, and sperm capacitation activities such as CATSPER3, GLRA1, PAFAH1B1, and SEMG2. However, boar SP protein members involved in the reproductive process function tended to be more associated with fertilization (4). The SP proteins related to spermatogenesis have been selected for use in the development of biomarkers for diagnosing invasively non-obstructive azoospermia (NOA) in humans (43). DDX4, recognized as a general germ cell marker present across all stages of germ cell development, including spermatogonia, spermatocytes, and round spermatids, has been chosen as one of the protein biomarkers in seminal plasma for diagnosing non-obstructive azoospermia (NOA) (44).

However, most of these mapped proteins involved in spermatogenesis or sperm capacitation were assessed for their roles in reproductive function based on their localization within the sperm cell. Analysis of expression microarrays conducted on testicular biopsy specimens from azoospermia patients revealed a significant downregulation of SPATA18 mRNA in biopsy specimens obtained from non-obstructive azoospermia (NOA) patients, in comparison to those collected from obstructive azoospermia (OA) patients (45). Moreover, SPATA18 protein is localized in satellite fibers that are associated with outer dense fibers within the midpiece of spermatozoa. The observed downregulation of SPATA18 in the asthenospermia group highlights the crucial role of this gene in sperm motility and fertility (46). Additionally, Asian elephant SP was discovered 19 zinc finger proteins (ZFPs) with various types, e.g., zinc finger C2H2-type, zinc finger CCCH-type, and zinc finger matrin-type. ZFP105, a C2H2-type ZFP, exhibited notable expression in the testes of adult mice, with its expression being regulated during postnatal development. Similar to other zinc finger proteins, ZFP105 is presumed to operate as an RNA-binding protein within spermatogenic cells, primarily spermatocytes, to exert its biological effects. Additionally, ZFP105 might serve as a cytoplasmic protein within spermatogenic cells, potentially engaging in direct or indirect interactions with other proteins implicated in the regulation of spermatogenesis and male fertility (47).

CATSPER, a sperm-specific ion channel, is vital for regulating sperm hyperactivation as a polymodal, chemosensory calcium channel. CATSPER3 and CATSPER4, recently identified as testis-specific genes, are crucial for sperm hyperactivated motility and male fertility in mice. Knockout of CATSPER3 and CATSPER4 in male mice leads to complete infertility due to rapid loss of motility and absence of hyperactivated motility under capacitating conditions (48). SMEG1 and SMEG2 are dominant proteins in human seminal plasma, contributing to the gel-like coagulum in ejaculated semen. They are produced by the glandular epithelium of the seminal vesicles in high concentrations. Seminal plasma motility inhibitors (SPMIs) are proteinase-resistant fragments derived from SMEG1 and SMEG2, which form the main proteins in the semen coagulum. A negative correlation was observed between sperm motility and the presence/intensity of SPMI labeling on spermatozoa (49).

Proteomic analysis of SP has been widely conducted in various animal species, particularly in agricultural mammal such as ram (11, 50), bull (7, 32), and boar (4, 31). Many SP proteins found in other species have also been identified in the Asian elephant, although some proteins exhibit different isoforms or subunits. For instance, aldose reductase, THBS1, and FN1, which were identified in boar SP (4, 31), were also presented in Asian elephant SP. However, keratin type I and aldosterone reductase in boar and Asian elephant SP exist in different subunits, such as KRT9, KRT15, and AVDP in Asian elephants, and KRT17 and AKR1B1 in boars (4). High expression of KRT17 and AKR1B1 in SP has been correlated with fertility rate in porcine, whereas boar semen with lower expression of THBS1 in SP appeared to produce larger litter sizes (4). Aldose reductase is an enzyme that plays a crucial role in sperm capacitation within the female reproductive tract of porcine (51). On the other hand, THBS1, an antiangiogenic protein, appears to have potential in the maternal-fetal interface during the early stages of porcine pregnancy (52). Furthermore, the level of FN1 in seminal plasma has been suggested as a potential biomarker for boar semen with good freezability (31) and has been reported to be correlated with the motility and fertilizing ability of human spermatozoa (53). In contrast, PAFA, an enzyme, has shown a negative association with freezability in Guzerat bull (32), but it is positively related to sperm motility in humans (54) and fertility in boars (54). The PAFA protein is known to function in stabilizing the sperm membrane during the process of sperm freezing (32).

Furthermore, the SP of Asian elephants contains several heparin-binding proteins described in ram SP, including BPI, HSP70, TPPP2, and MANB1, although in different subunits, except for PGK2 (11). BPI, an antimicrobial protein, functions by damaging the membrane of gram-negative bacteria through binding to lipopolysaccharide and activating endogenous phospholipase (56). Chaperone protein families such as HSP have been reported to be involved in spermatogenesis and the preparation of the sperm membrane before interfacing with the zona pellucida of the oocyte (30). Additionally, HSP60, which acts as a signaling molecule within the immune system, interacts with glutathione peroxidase, a scavenger of reactive oxygen species, to protect sperm membrane from lipid peroxidation (32). TPPP family members play a role in maintaining the integrity of microtubules in ciliated cells, including neurons and sperm cells, suggesting a possible involvement in sperm tail stabilization (11). Mannosidase, an enzyme originating from the epididymis, is found in the seminal plasma of various species (7, 57, 58), including Asian elephants. Mannosidase is also presented on the sperm membrane and is involved in sperm-egg fertilization (11).

Thirteen proteins out of the 99 proteins in the SP proteome of Bull (*Bos Taurus*) reported by Kelly et al. (7) were found in this proteomic analysis of Asian elephant SP. However, half of these similar proteins, such as PAFA, DEFB, TCP1, and TEX, were different isoforms or subunits. DEFBs, which are host defense peptides, are known to be expressed in both male and female reproductive tracts and are also found on sperm surface. They are believed to play a role in fertility and sperm function by protecting spermatozoa from infections through their antimicrobial activity and preventing premature hyperactivation of spermatozoa within the female reproductive tract (59). The distal inversion of the T-complex on chromosome 17 comprises genes that specifically impact male fertility. Furthermore, a number of proteins encoded by these t-complex genes are specifically found in the sperm flagellum and are associated with various functions related to sperm motility (60). CCT2 (TCP1 beta subunit), which was found in Asian elephant SP, has been identified in the flagella of the sperm tail in humans (61) and is involved in sperm maturation in epididymis (62).

## Conclusion

Several proteins discovered in Asian elephant seminal plasma (SP) have been reported to be involved in reproductive function and have shown potential as biomarkers for fertility, sperm quality, and preservation. However, a significant number of these proteins are predicted to have their primary impact as proteins derived from sperm cells rather than SP proteins. Therefore, when investigating male reproductive problems, it is crucial to consider the comparison between protein expression in spermatozoa and SP proteins in order to obtain a comprehensive understanding of the root causes of these issues. Although various SP proteins in Asian elephants have been matched with SP proteins from other species, most of them differ in terms of isoforms or subunits. The exact functions of these Asian elephant SP proteins still need to be explored.

## Data availability statement

The datasets presented in this study can be found in online repositories. The names of the repository/repositories and accession number(s) can be found in the article/Supplementary material.

## Ethics statement

The animal study was reviewed and approved by Institutional Animal Care and Use Committee, Kasetsart University (VETKU-IACUC). Written informed consent was obtained from the owners for the participation of their animals in this study.

## Author contributions

PW, SR, and AP contributed to experimental design of this research. SM organized the sample collection. PW, KK, and AP collected samples and performed semen evaluation. PW and YY manipulated and analyzed proteins from samples. PW performed the statistical analysis and wrote the first draft of the manuscript. All authors contributed to the article and approved the submitted version.

## Funding

This research was supported by the Center for Advanced Studies for Agriculture and Food (CASAF), Kasetsart University, Bangkok, Thailand.

## Conflict of interest

The authors declare that the research was conducted in the absence of any commercial or financial relationships that could be construed as a potential conflict of interest.

## Publisher’s note

All claims expressed in this article are solely those of the authors and do not necessarily represent those of their affiliated organizations, or those of the publisher, the editors and the reviewers. Any product that may be evaluated in this article, or claim that may be made by its manufacturer, is not guaranteed or endorsed by the publisher.

## Footnotes

^1^ http://proteomecentral.proteomexchange.org

^2^ https://jpostdb.org

^3^ http://www.pantherdb.org/

## References

[1] Hildebrandt TB Goeritz F Hermes R Reid C Dehnhard M Brown JL. Aspects of the reproductive biology and breeding Management of Asian and African Elephants: Elephas Maximus and Loxodonta Africana. Int Zoo Yb. (2006) 40:20–40. doi: 10.1111/j.1748-1090.2006.00020.x

[2] Thitaram C. Breeding Management of Captive Asian Elephant (*Elephas Maximus*) in range countries and zoos. Jpn J Zoo Wildl Med. (2012) 17:91–6. doi: 10.5686/jjzwm.17.91

[3] Wattananit P. Refining Asian elephant semen technologies: The need for improved quality analysis and preservation techniques. Utrecht, The Netherlands: Urecht University (2014).

[4] Perez-Patino C Parrilla I Barranco I Vergara-Barberan M Simo-Alfonso EF Herrero-Martinez JM. New in-depth analytical approach of the porcine seminal plasma proteome reveals potential fertility biomarkers. J Proteome Res. (2018) 17:1065–76. doi: 10.1021/acs.jproteome.7b00728, PMID: 29411616

[5] Bianchi L Carnemolla C Viviani V Landi C Pavone V Luddi A. Soluble protein fraction of human seminal plasma. J Proteome. (2018) 174:85–100. doi: 10.1016/j.jprot.2017.12.015, PMID: 29288815

[6] Aquino-Cortez A Pinheiro BQ Lima DBC Silva HVR Mota-Filho AC Martins JAM. Proteomic characterization of canine seminal plasma. Theriogenology. (2017) 95:178–86. doi: 10.1016/j.theriogenology.2017.03.016, PMID: 28460673

[7] Kelly VC Kuy S Palmer DJ Xu Z Davis SR Cooper GJ. Characterization of bovine seminal plasma by proteomics. Proteomics. (2006) 6:5826–33. doi: 10.1002/pmic.200500830 17001600

[8] Strzezek J Wysocki P Kordan W Kuklinska M Mogielnicka M Soliwoda D. Proteomics of boar seminal plasma—current studies and possibility of their application in biotechnology of animal reproduction. Reprod Biol. (2005) 5:279–90. PMID: 16372045

[9] Nowicka-Bauer K Kurpisz M. Current knowledge of the human sperm proteome. Expert Rev Proteomics. (2013) 10:591–605. doi: 10.1586/14789450.2013.846221, PMID: 24168729

[10] Intasqui P Camargo M Antoniassi MP Cedenho AP Carvalho VM Cardozo KHM. Association between the seminal plasma proteome and sperm functional traits. Fertil Steril. (2016) 105:617–28. doi: 10.1016/j.fertnstert.2015.11.005 26621572

[11] Martins JAM Souza CEA Silva FDA Cadavid VG Nogueira FC Domont GB. Major heparin-binding proteins of the seminal plasma from Morada Nova rams. Small Rumin Res. (2013) 113:115–27. doi: 10.1016/j.smallrumres.2013.01.005

[12] Thongtip N Saikhun J Mahasawangkul S Kornkaewrat K Pongsopavijitr P Songsasen N. Potential factors affecting semen quality in the Asian elephant (*Elephas Maximus*). Reprod Biol Endocrinol. (2008) 6:9. doi: 10.1186/1477-7827-6-9, PMID: 18346275 PMC2276508

[13] Kiso WK Selvaraj V Nagashima J Asano A Brown JL Schmitt DL. Lactotransferrin in Asian elephant (*Elephas Maximus*) seminal plasma correlates with semen quality. PLoS One. (2013) 8:e71033. doi: 10.1371/journal.pone.0071033, PMID: 23976974 PMC3745378

[14] Negus C. Liquid and frozen storage of Asian elephant spermatozoa. Sydney, Australia: The University of Sydney (2021).

[15] Kumar P Kumar D Singh I Yadav PS. Seminal plasma proteome: promising biomarkers for bull fertility. Agric Res. (2012) 1:78–86. doi: 10.1007/s40003-011-0006-2

[16] Sobsey CA Ibrahim S Richard VR Gaspar V Mitsa G Lacasse V. Targeted and untargeted proteomics approaches in biomarker development. Proteomics. (2020) 20:e1900029. doi: 10.1002/pmic.201900029, PMID: 31729135

[17] Medzihradszky KF. In-solution digestion of proteins for mass spectrometry. Methods Enzymol. (2005) 405:50–65. doi: 10.1016/S0076-6879(05)05003-2 16413310

[18] Wisniewski JR Zougman A Nagaraj N Mann M. Universal sample preparation method for proteome analysis. Nat Methods. (2009) 6:359–62. doi: 10.1038/nmeth.1322 19377485

[19] Schmitt DL Hildebrandt TB. Manual collection and characterization of semen from Asian elephants (*Elephas Maximus*). Anim Reprod Sci. (1998) 53:309–14. doi: 10.1016/s0378-4320(98)00120-1 9835384

[20] Bjorndahl L Soderlund I Kvist U. Evaluation of the one-step eosin-Nigrosin staining technique for human sperm vitality assessment. Hum Reprod. (2003) 18:813–6. doi: 10.1093/humrep/deg199, PMID: 12660276

[21] Sarder MJU. Effects of age, body weight, body condition and scrotal circumference on sperm abnormalities of bulls used for artificial insemination (Ai) Programme in Bangladesh university. J Zool Rajshahi Univ. (2008) 27:73–8. doi: 10.3329/ujzru.v27i0.1959

[22] Lowry OH Rosebrough NJ Farr AL Randall RJ. Protein measurement with the Folin phenol reagent. J Biol Chem. (1951) 193:265–75. doi: 10.1016/S0021-9258(19)52451-6 14907713

[23] Leon IR Schwammle V Jensen ON Sprenger RR. Quantitative assessment of in-solution digestion efficiency identifies optimal protocols for unbiased protein analysis. Mol Cell Proteomics. (2013) 12:2992–3005. doi: 10.1074/mcp.M112.025585, PMID: 23792921 PMC3790306

[24] Johansson C Samskog J Sundstrom L Wadensten H Bjorkesten L Flensburg J. Differential expression analysis of *Escherichia Coli* proteins using a novel software for relative quantitation of LC-MS/MS data. Proteomics. (2006) 6:4475–85. doi: 10.1002/pmic.200500921, PMID: 16858737

[25] Thorsell A Portelius E Blennow K Westman-Brinkmalm A. Evaluation of sample fractionation using Micro-scale liquid-phase isoelectric focusing on mass spectrometric identification and quantitation of proteins in a Silac experiment. Rapid Commun Mass Spectrom. (2007) 21:771–8. doi: 10.1002/rcm.2898, PMID: 17279600

[26] Perkins DN Pappin DJ Creasy DM Cottrell JS. Probability-based protein identification by searching sequence databases using mass spectrometry data. Electrophoresis. (1999) 20:3551–67. doi: 10.1002/(SICI)1522-2683(19991201)20:18<3551::AID-ELPS3551>3.0.CO;2-2 10612281

[27] Consortium TU. Uniprot: the universal protein knowledgebase in 2021. Nucleic Acids Res. (2020) 49:D480–9. doi: 10.1093/nar/gkaa1100, PMID: 33237286 PMC7778908

[28] Thomas PD Campbell MJ Kejariwal A Mi H Karlak B Daverman R. Panther: a library of protein families and subfamilies indexed by function. Genome Res. (2003) 13:2129–41. doi: 10.1101/gr.772403, PMID: 12952881 PMC403709

[29] Szklarczyk D Kirsch R Koutrouli M Nastou K Mehryary F Hachilif R. The string database in 2023: protein-protein association networks and functional enrichment analyses for any sequenced genome of interest. Nucleic Acids Res. (2023) 51:D638–46. doi: 10.1093/nar/gkac1000, PMID: 36370105 PMC9825434

[30] Pini T Leahy T Soleilhavoup C Tsikis G Labas V Combes-Soia L. Proteomic investigation of ram spermatozoa and the proteins conferred by seminal plasma. J Proteome Res. (2016) 15:3700–11. doi: 10.1021/acs.jproteome.6b00530, PMID: 27636150

[31] Vilagran I Yeste M Sancho S Castillo J Oliva R Bonet S. Comparative analysis of boar seminal plasma proteome from different Freezability ejaculates and identification of fibronectin 1 as sperm Freezability marker. Andrology. (2015) 3:345–56. doi: 10.1111/andr.12009, PMID: 25678437

[32] Rego JP Martins JM Wolf CA van Tilburg M Moreno F Monteiro-Moreira AC. Proteomic analysis of seminal plasma and sperm cells and their associations with semen Freezability in Guzerat bulls. J Anim Sci. (2016) 94:5308–20. doi: 10.2527/jas.2016-0811 28046165

[33] Lundwall A Bjartell A Olsson AY Malm J. Semenogelin I and II, the predominant human seminal plasma proteins, are also expressed in non-genital tissues. Mol Hum Reprod. (2002) 8:805–10. doi: 10.1093/molehr/8.9.805, PMID: 12200457

[34] Viana AGA Martins AMA Pontes AH Fontes W Castro MS Ricart CAO. Proteomic landscape of seminal plasma associated with dairy bull fertility. Sci Rep. (2018) 8:16323. doi: 10.1038/s41598-018-34152-w, PMID: 30397208 PMC6218504

[35] Fu Q Pan L Huang D Wang Z Hou Z Zhang M. Proteomic profiles of Buffalo spermatozoa and seminal plasma. Theriogenology. (2019) 134:74–82. doi: 10.1016/j.theriogenology.2019.05.013, PMID: 31146187

[36] Milardi D Grande G Vincenzoni F Castagnola M Marana R. Proteomics of human seminal plasma: identification of biomarker candidates for fertility and infertility and the evolution of technology. Mol Reprod Dev. (2013) 80:350–7. doi: 10.1002/mrd.22178, PMID: 23559416

[37] Batruch I Lecker I Kagedan D Smith CR Mullen BJ Grober E. Proteomic analysis of seminal plasma from Normal volunteers and post-vasectomy patients identifies over 2000 proteins and candidate biomarkers of the urogenital system. J Proteome Res. (2011) 10:941–53. doi: 10.1021/pr100745u, PMID: 21142078

[38] Milardi D Grande G Vincenzoni F Messana I Pontecorvi A De Marinis L. Proteomic approach in the identification of fertility pattern in seminal plasma of fertile men. Fertil Steril. (2012) 97:67–73.e1. doi: 10.1016/j.fertnstert.2011.10.013, PMID: 22088208

[39] Camargo M Intasqui P Bertolla RP. Understanding the seminal plasma proteome and its role in male fertility. Basic Clin Androl. (2018) 28:6. doi: 10.1186/s12610-018-0071-5, PMID: 29881623 PMC5985566

[40] Lazar J Rasmussen L Greenwood DR Bang I-S Prestwich GD. Elephant albumin: a multipurpose pheromone shuttle. Chem Biol. (2004) 11:1093–100. doi: 10.1016/j.chembiol.2004.05.018 15324810

[41] Hashiguchi Y. The origin and evolution of the trace amine-associated receptor family in vertebrates. In: eds. Farooqui T. Farooqui A. A. Trace Amines Neurol Disord. London, UK: Elsevier. (2016):45–62. doi: 10.1016/B978-0-12-803603-7.00004-5

[42] Staubert C Boselt I Bohnekamp J Rompler H Enard W Schoneberg T. Structural and functional evolution of the trace amine-associated receptors Taar3, Taar4 and Taar5 in Primates. PLoS One. (2010) 5:e11133. doi: 10.1371/journal.pone.0011133, PMID: 20559446 PMC2886124

[43] Araujo SC Bertolla RP. Protein markers of spermatogenesis and their potential use in the Management of Azoospermia. Expert Rev Proteomics. (2021) 18:939–48. doi: 10.1080/14789450.2021.2010548, PMID: 34812697

[44] Abdallah W Hashad D Abdelmaksoud R Hashad MM. Does detection of Ddx 4 Mrna in cell-free seminal plasma represents a reliable noninvasive germ cell marker in patients with nonobstructive azoospermia? Andrologia. (2017) 49:e12739. doi: 10.1111/and.12739 28000927

[45] Bornstein C Brosh R Molchadsky A Madar S Kogan-Sakin I Goldstein I. Spata18, a spermatogenesis-associated gene, is a novel transcriptional target of P53 and P63. Mol Cell Biol. (2011) 31:1679–89. doi: 10.1128/MCB.01072-10 21300779 PMC3126342

[46] Panahi A Ahmadi SM Tehrani GA. Comparison between Spata18 and P53 gene expressions in the sperm cells obtained from Normospermic and Asthenospermic samples: a case-control study. Int J Fertil Steril. (2022) 16:122–7. doi: 10.22074/IJFS.2021.138190.1029, PMID: 35639655 PMC9108294

[47] Zhou H Liu LH Zhang H Lei Z Lan ZJ. Expression of zinc finger protein 105 in the testis and its role in male fertility. Mol Reprod Dev Incorp Gam Res. (2010) 77:511–20. doi: 10.1002/mrd.21171, PMID: 20186958 PMC3062163

[48] Jin J Jin N Zheng H Ro S Tafolla D Sanders KM. Catsper3 and Catsper4 are essential for sperm Hyperactivated motility and male fertility in the mouse. Biol Reprod. (2007) 77:37–44. doi: 10.1095/biolreprod.107.060186, PMID: 17344468

[49] Terai K Yoshida K Yoshiike M Fujime M Iwamoto T. Association of Seminal Plasma Motility Inhibitors/Semenogelins with sperm in Asthenozoospermia-infertile men. Urol Int. (2010) 85:209–15. doi: 10.1159/000315976, PMID: 20720384

[50] Sousa FM Lobo CH Menezes ES Rego JP Oliveira RV Lima-Souza AC. Parameters of the reproductive tract, spermatogenesis, daily sperm production and major seminal plasma proteins of tropically adapted Morada Nova rams. Reprod Domest Anim. (2014) 49:409–19. doi: 10.1111/rda.12288, PMID: 24716618

[51] Katoh Y Takebayashi K Kikuchi A Iki A Kikuchi K Tamba M. Porcine sperm capacitation involves tyrosine phosphorylation and activation of aldose reductase. Reproduction. (2014) 148:389–401. doi: 10.1530/REP-14-0199, PMID: 25049426

[52] Edwards AK van den Heuvel MJ Wessels JM Lamarre J Croy BA Tayade C. Expression of Angiogenic basic fibroblast growth factor, platelet derived growth factor, Thrombospondin-1 and their receptors at the porcine maternal-fetal Interface. Reprod Biol Endocrinol. (2011) 9:5. doi: 10.1186/1477-7827-9-5, PMID: 21241502 PMC3032667

[53] Wennemuth G Meinhardt A Mallidis C Albrecht M Krause W Renneberg H. Assessment of fibronectin as a potential new clinical tool in andrology. Andrologia. (2001) 33:43–6. doi: 10.1046/j.1439-0272.2001.00370.x, PMID: 11167518

[54] Roudebush WE Purnell ET. Platelet-activating factor content in human spermatozoa and pregnancy outcome. Fertil Steril. (2000) 74:257–60. doi: 10.1016/s0015-0282(00)00646-4 10927041

[55] Roudebush WE Diehl JR. Platelet-activating factor content in boar spermatozoa correlates with fertility. Theriogenology. (2001) 55:1633–8. doi: 10.1016/s0093-691x(01)00508-8, PMID: 11396471

[56] Gabrielli NM Veiga MF Matos ML Quintana S Chemes H Blanco G. Expression of Dysadherin in the human male reproductive tract and in spermatozoa. Fertil Steril. (2011) 96:554–561.e2. doi: 10.1016/j.fertnstert.2011.06.053, PMID: 21774927

[57] Dzugan M Ksiazkiewicz J. Activity of alpha- and Beta-mannosidases in semen and reproductive organs of the drake. Reprod Biol. (2009) 9:25–37. doi: 10.1016/S1642-431X(12)60092-8, PMID: 19352415

[58] Jauhiainen A Vanha-Perttula T. Characterization of acid and neutral alpha-mannosidases in bull semen and reproductive organs. Int J Biochem. (1987) 19:267–74. doi: 10.1016/0020-711x(87)90030-9, PMID: 3595978

[59] Dorin JR Barratt CL. Importance of Beta-Defensins in sperm function. Mol Hum Reprod. (2014) 20:821–6. doi: 10.1093/molehr/gau050 25009294

[60] Liu Y Jiang M Li C Yang P Sun H Tao D. Human T-complex protein 11 (Tcp11), a testis-specific gene product, is a potential determinant of the sperm morphology. Tohoku J Exp Med. (2011) 224:111–7. doi: 10.1620/tjem.224.111 21597245

[61] Baker MA Naumovski N Hetherington L Weinberg A Velkov T Aitken RJ. Head and flagella subcompartmental proteomic analysis of human spermatozoa. Proteomics. (2013) 13:61–74. doi: 10.1002/pmic.201200350, PMID: 23161668

[62] Dacheux JL Belleannee C Guyonnet B Labas V Teixeira-Gomes AP Ecroyd H. The contribution of proteomics to understanding Epididymal maturation of mammalian spermatozoa. Syst Biol Reprod Med. (2012) 58:197–210. doi: 10.3109/19396368.2012.663233, PMID: 22788532

